# Supplementary material for: Peripheral-specific Y1 receptor antagonism increases thermogenesis and protects against diet-induced obesity
Source: Nat Commun. 2021 May 11;12:2622. doi: 10.1038/s41467-021-22925-3 (PMC8113522; doi:10.1038/s41467-021-22925-3)
Supplement: Supplementary file 1 — Supplementary Information File [file 41467_2021_22925_MOESM1_ESM.pdf]

Supplementary information to manuscript entitled "Peripheral-specific Y1 receptor antagonism increases thermogenesis and protects against diet-induced obesity" by Yan et al. Nature Communications 2021

Supplementary Table 1. Basic characteristics of lean and obese subjects

	Men	Women	Age (Years)	BMI (kg/m2)	HR (bpm)	BP (mmHg)	TG (mmol/L)	TC (mmol/L)
Lean subjects	6	5	39.72±11.76	20.75±1.98	75.5±11	120±8/75±7	0.99±0.28	3.88±0.85
Obese subjects	8	8	34.75±8.86	41.52±7.55	87.4±9	132±15/83±13	2.25±0.91	4.99±1.47

Supplementary Table 2. Sequences of oligonucleotide primers (5'-3') used in real time PCR in humans

Gene symbol	5' Oligonucleotide	3' Oligonucleotide
NPY	CGCTGCGACACTACATCAAC	CTCTGGGCTGGATCGTTTTCC
UCP1	GATCACCTTCCCGCTGGACA	AGCTGATTTGCCGCTGAAGC
PGC1α	ACCACAAACGATGACCCTCC	GCCTGCAGTTCCAGAGAGTT
Cidea	CTTGGGAGACAACACGCATTT	TCTCGCTATTCCCGACCTCTT
CD137	TTGGATGGAAAGTCTGTGCTTG	AGGAGATGATCTGCGGAGAGT
Tmem26	TTTGCCAGTACAGTGCCGAT	ACCACCAAGCGGTAGAGTTG
Adiponectin	GACCAGGAAACCACGACTCA	TAGGCAAAGTAGTACAGCCCA
PPARγ	TACTGTCGGTTTCAGAAATGCC	GTCAGCGGACTCTGGATTCAG
NPYR1(Y1R)	GAGGCGATGTGTAAGTTGAATCC	TGGAACGGCTCATCAGTCATT
NPYR2(Y2R)	CATCTTGCTTGGGGTAATTGGC	AGAGTGAACGGTAGACACAGAG
NPYR4(Y4R)	TCCAGTGCATGTCGGTGAC	ACACAGGCAATGACCCAGATG
NPYR5(Y5R)	GCTGGATCAGTGGATGTTTGG	CAGATGGCAAAACCTAGTGTCC

Supplementary Table 3. Sequences of oligonucleotide primers (5'-3') used in real-time PCR in mice

Gene symbol	5' Oligonucleotide	3' Oligonucleotide
NPYR1(Y1R)	CACAGGCTGTCTTACACG	GCGAATGTATATCTTGAAGTAG
NPYR2(Y2R)	CACCAAATCGGACCTGCT	AGAACCAGTTCACTCTCACTTGG
NPYR4(Y4R)	CTCCTCACCTGGGACGTG	TCCAGGAGTAAAGGGCCTACT
NPYR5(Y5R)	GAGAAGCCCACGTCCCTATC	AGAGTCCCTCTTGGAAACCAC
UCP1	GGCCTCTACGACTCAGTCCA	TAAGCCGGCTGAGATCTTGT
PGC1α	CAGTCGCAACATGCTCAAG	GGGTCATTTGGTGACTCTGG
PRDM16	CCTAAGGTGTGCCCAGCA	CACCTTCCGCTTTTCTACCC
DIO2	CTGCGCTGTGTCTGGAAC	GGAGCATCTTCACCCAGTTT
Tbx1	TTTGTGCCCGTAGATGACAA	CTCGGCCAGGTGTAGCAG
Cox7a1	CGAAGAGGGGAGGTGACTC	AGCCTGGGAGACCCGTAG
NRG4	TGATTTTCAACCTTAATTCTTCCAT	CCCCCATTGAGGCAAAAT
CIDEA	CTCCGAGTACTGGGCGATAC	ACCAGCCTTTGGTGCTAGG
EVA1	TGTGCTTCCACTTCTCCTGA	TCCACAGCTTCTGTAGGACAAA
Slc27a1	GACAAGCTGGATCAGGCAAG	GAGGCCACAGAGGCTGTTC
Acot2	GGAACCGAGGGCTGAAGT	GCTCTCAGGACAGCGAAAGA
Tmem26	GTGTTTCAATAAAGTATTTGGACAGC	TTAAGCCAGATTTTGGACAGG
PDK4	TCAAGATTTCTGACCGAGGAG	TGTAACTAAAGAGGCGGTCAGTAA
Slc29a1	CCTGCAGCTCAACCTTGC	CTTCCTTTTGGCTCCTCTCTT
Klhl13	GTGGCCAGAGCTTTCACAG	GCCTTAGGCCACAGCTAATAGA
FBXO31	TGGCTTGCTTAGATCTTGGAC	GCTCCTAGCAACCAGGACAG
Ebf3	GCACAACAATTCCAAACACG	GGGGCCGTACCTTCTGAC
CD137	GGCCTTCCAGTCCACCAT	GTCCAGGAGTCATGCAGAGG
IRF4	AGCACCTTATGGCTCTCTGC	TGACTGGTCAGGGGCATAAT

PPARγ	GAAAGACAACGGACAAATCACC	GGGGGTGATATGTTTGAACTTG
Adiponectin	GGAGAGAAAGGAGATGCAGGT	CTTTCCTGCCAGGGGTTC
β3R	CAGCCAGCCCTGTTGAAG	CCTTCATAGCCATCAAACCTG
NRG4	TGATTTTCAACCTTAATTCTTCCAT	CCCCCATTGAGGCAAAAT
RPL19	CTCGTTGCCGGAAAAACA	TCATCCAGGTCACCTTCTCA

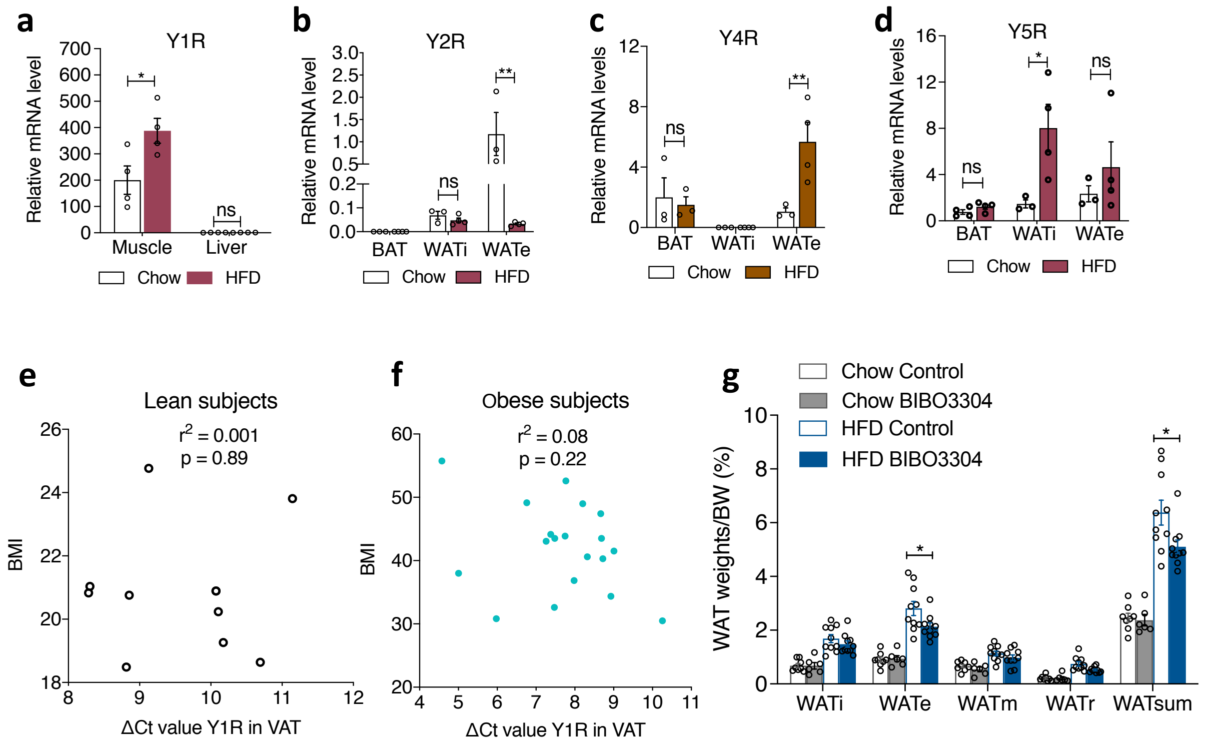

Supplementary Fig 1: mRNA expression profile of other NPY receptors in mice and human adipose tissues. **a**, *Y1R* mRNA expression in muscle and liver; **b**, *Y2R* mRNA expression, **c**, *Y4R* mRNA expression, **d**, *Y5R* mRNA expression in BAT, WATi and WATe of wild type mice fed a standard chow diet (open bar) or a HFD (filled bar) for 7 weeks. Data are mean ± s.e.m. n = 3-4 per group. **p* < 0.05; ***p* < 0.01, two-tailed t test within the same tissue type. ns, non-significance. **e, f**, Correlation between BMI (kg/m^2) and ΔCt values of *Y1R* mRNA expression in visceral adipose tissue (VAT) of lean subjects (left) and obese subjects (right). Data are mean ± s.e.m. n = 11 in lean individuals and n = 16 in obese individuals. *p* values by two tailed Pearson Correlation analysis. **g**, Dissected white adipose tissue weights expressed as a percent of body weight of chow- or HFD-fed WT mice treated with control (open bar) or BIBO3304 (filled bar) for 7 weeks. Data are mean ± s.e.m, chow n = 6 (grey, control: open grey; BIBO3304: grey), HFD n = 8 (control: open blue; BIBO3304: blue), **p* < 0.05, two-way ANOVA with Sidak's multiple comparisons test. Source data are provided as a Source Data file.

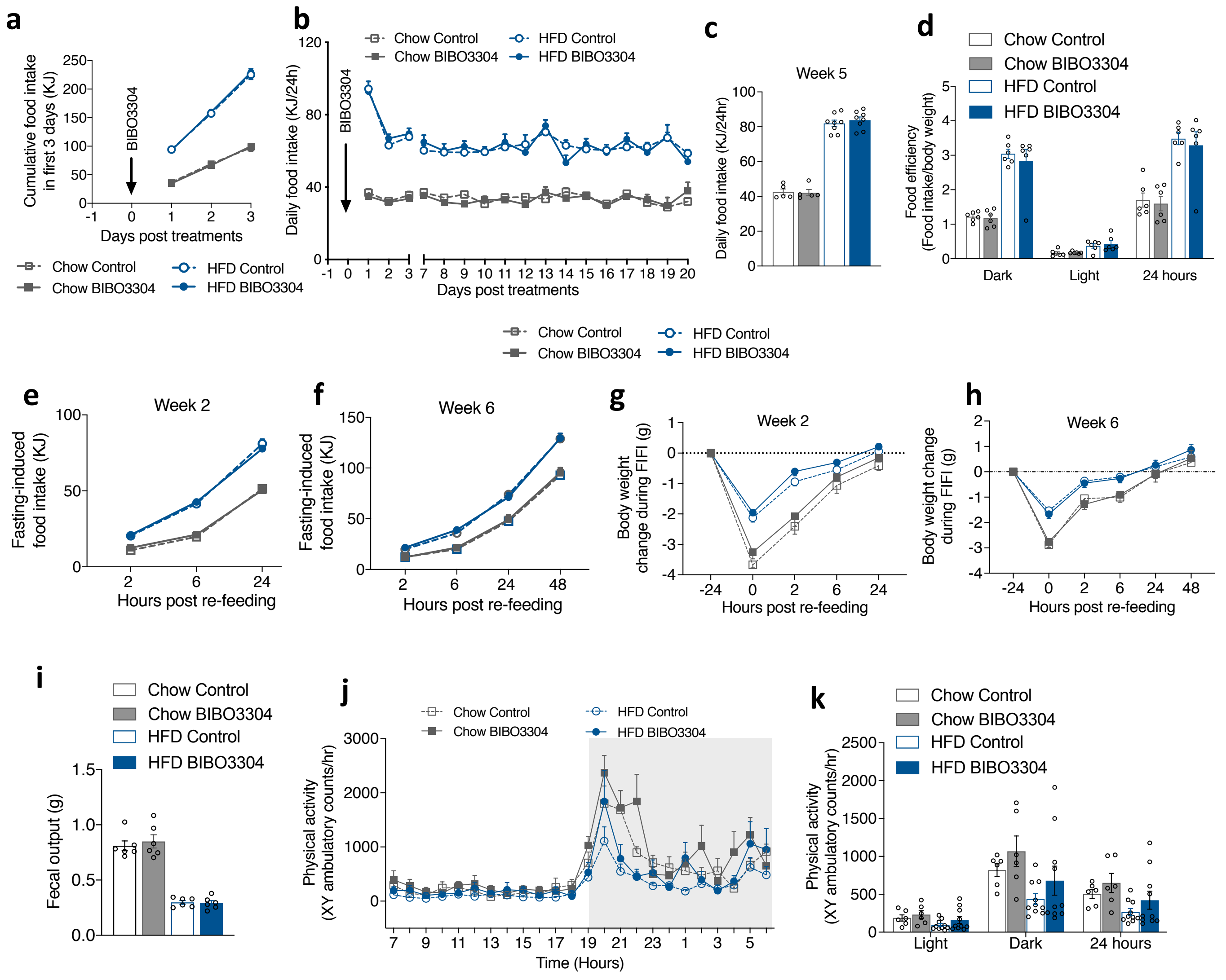

Supplementary Fig. 2: Effect of peripheral Y1R antagonism on food intake and energy expenditure in wild type mice. **a**, Cumulative food intake of chow- and HFD-fed WT mice treated with a vehicle or BIBO3304 containing jelly in first 3 days. **b**, Daily food intake of chow- and HFD-fed WT mice treated with a vehicle or BIBO3304 containing jelly from day 1 to day 20 post treatment. **c**, Food intake of chow- and HFD-fed WT mice treated with a vehicle or BIBO3304 containing jelly at week 5 post treatment. Data are mean ± s.e.m, chow n = 5 (control: open grey; BIBO3304: grey), HFD n = 8 (control: open blue; BIBO3304: blue), *p* values by repeated measures ANOVA (**a**, **b**) or two-way ANOVA (**c**). **d**, Food efficiency was calculated during the dark phase, light phase and over a 24-h period. *p* value by two-tailed t-test within the same adipose depot. Data are mean ± s.e.m, n = 6 per group, *p* values by two-way ANOVA with Sidak's multiple comparisons test. **e, f**, Fast-induced food intake performed at week 2 and week 6 post BIBO3304 treatment. **g, h**, Weight recovery after the fast-induced food intake at week 2 and week 6 in **e, f**. Data are mean ± s.e.m, chow n = 6 (grey, control: open square; BIBO3304: filled square), HFD n = 8 (blue, control: open circle; BIBO3304: filled circle), *p* values by two-way repeated measures ANOVA (**e**, **f**, **g**, **h**). **i**, Fecal output of chow- and HFD-fed mice treated with vehicle or BIBO3304 for 7 weeks. Data are mean ± s.e.m, chow n = 6, HFD n = 8, *p* values by two-way ANOVA with Sidak's multiple comparisons test. **j,** Physical activity over a 24h course, with **k** showing average physical activity during the light phase, dark phase and over a 24 h period from **j**. Data are mean ± s.e.m, chow n = 6, HFD n = 8, *p* values by two-way repeated measures ANOVA with Sidak's multiple comparisons test. Source data are provided as a Source Data file.

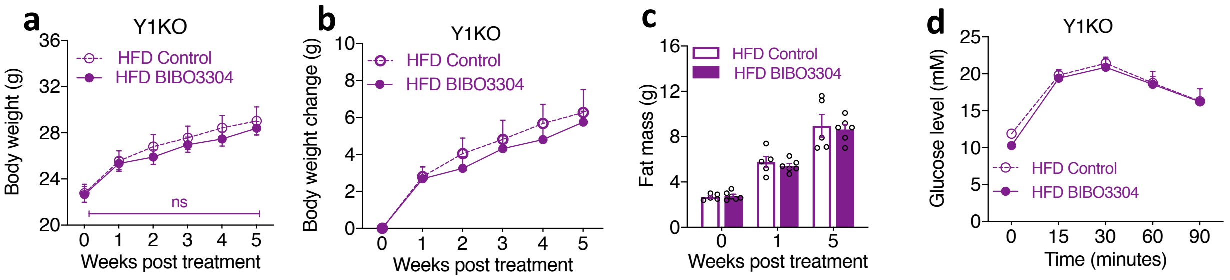

Supplementary Fig. 3: Y1R signalling is critical for BIBO3304-induced thermogenic effects. BIBO3304-treated HFD-fed Y1R$^{-/-}$ mice displayed no difference from control treated Y1R$^{-/-}$ mice with regards to (**a**) body weight, (**b**) body weight change (control: open circle; BIBO3304: filled circle), (**c**) whole-body fat mass (control: open bar; BIBO3304: filled bar) and (**d**) glucose tolerance test. Data are mean ± s.e.m, n = 4 -6 per group. *p* values by two-way ANOVA or repeated measures (**a, b, d**) with Sidak's multiple comparisons test, two-sided t test for fat mass (**c**). ns, non-significance. Source data are provided as a Source Data file.

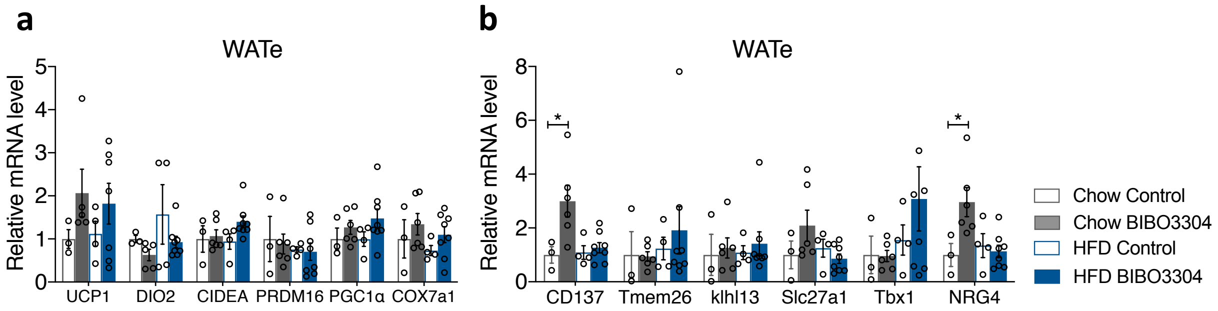

Supplementary Fig. 4: mRNA expression of BAT-specific and beige-selective genes in epididymal white adipose tissue (WATe) was unaffected by BIBO3304 treatment. **a**, mRNA levels of thermogenic markers in WATe. **b**, mRNA levels of beige adipocyte-related genes in WATe of chow- and HFD-fed wild type mice on a daily administration with a jelly containing a vehicle or BIBO3304. Data are mean ± s.e.m, chow n =3 – 6 (control: open grey; BIBO3304: grey), HFD n = 4 – 8 (control: open blue; BIBO3304: blue). *$p < 0.05$, two-way ANOVA with Sidak's multiple comparisons test. Source data are provided as a Source Data file.

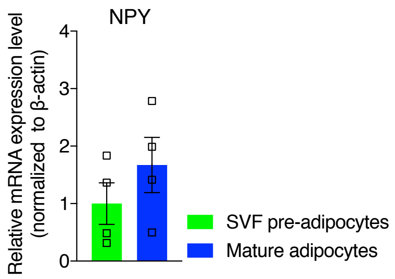

Supplementary Fig. 5. NPY mRNA expression in human SVF pre-adipocytes and mature adipocytes. Data are mean ± s.e.m, n = 4 subjects per group (pre-adipocytes: bright green; mature adipocyte: blue). *p* values by two-sided unpaired t test. Source data are provided as a Source Data file.

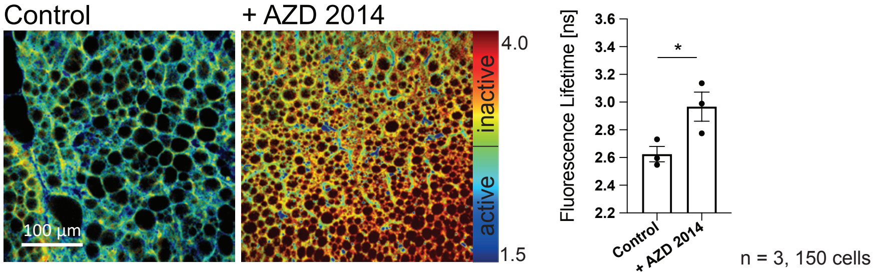

Supplementary Fig. 6. Akt activity in brown adipose tissue was blocked by specific Akt inhibitor in Akt-FRET biosensor mice. Brown adipose tissue (BAT) isolated from chow-fed Akt-FRET biosensor mice was treated *ex vivo* with 500 nM AZD2014 (a mTOR1/2 inhibitor) for 30 minutes and Akt activity quantified, revealing effective inhibition of Akt in this tissue. Data are mean± s.e.m. n = 3 mice per group, 150 cells; $*p < 0.05$, two-tailed unpaired t test. Source data are provided as a Source Data file.
